# Ca(BF_4_)_2_·*x*H_2_O redefined from powder diffraction as hy­dro­gen-bonded Ca(H_2_O)_4_(BF_4_)_2_ ribbons

**DOI:** 10.1107/S2053229625004395

**Published:** 2025-05-19

**Authors:** Armel Le Bail

**Affiliations:** a Le Mans Université, Institut des Molécules et des Matériaux du Mans, CNRS UMR 6283, Av. Olivier Messiaen, 72085 Le Mans, France; Oak Ridge National Laboratory, USA

**Keywords:** fluoro­borate, calcium, hydrate, powder diffraction, *ab initio*

## Abstract

The crystal structure of the calcium bis(tetra­fluoro­borate) hydrate Ca(BF_4_)_2_·*x*H_2_O has been determined from laboratory powder diffraction data. The water mol­ecules all belong to [CaO_4_F_4_] square anti­prisms sharing F corners with [BF_4_] tetra­hedra, forming a mono-dimensional structure of infinite ribbons inter­connected by H⋯F and H⋯O hy­dro­gen bonds. No place is found for inter­stitial water mol­ecules, so that the com­pound has to be reformulated as Ca(H_2_O)_4_(BF_4_)_2_, which is isostructural with calcium perchlorate tetra­hydrate, Ca(ClO_4_)_2_·4H_2_O.

## Abstract 2

The crystal structure of Ca(BF_4_)_2_·*x*H_2_O has been solved *ab initio* and refined from laboratory powder diffraction data. The water mol­ecules all belong to [CaO_4_F_4_] square anti­prisms sharing F corners with [BF_4_] tetra­hedra, forming a mono-dimensional structure of infinite ribbons inter­connected by H⋯F and H⋯O hy­dro­gen bonds.

## Introduction

Calcium-based rechargeable batteries were thought to be impossible until the demonstration of the feasibility of calcium plating at moderate tem­per­a­tures (Ponrouch *et al.*, 2016 ▸). It was observed that optimal Ca metal deposition occurred using electrolytes containing Ca(BF_4_)_2_ in a mixture of ethyl­ene carbonate and propyl­ene carbonate at *T* > 75 °C. There was then a need for dry and contaminant-free Ca(BF_4_)_2_. Different synthetic routes were explored as alternatives to the drying of the commercial hydrated salt Ca(BF_4_)_2_·*x*H_2_O which proved to be not trivial by Forero-Saboya *et al.* (2020 ▸), who proposed a value for *x* of 4.6, estimated by Karl–Fisher coulometer titration. However, this would correspond to 28 wt%, and a two-step decom­position is observed during thermogravimetric analysis (TGA), at 158 and 240 °C, with losses of 14.3 and 52.5 wt%, respectively. Close to two water mol­ecules would escape first and it is believed that the remaining water persists in the solid and participates in the anion hydrolysis at tem­per­a­tures above 170 °C. An older estimation for *x* (= 5) can be found in the PDF card 00-022-0523, dated 1969 (Kabekkodu *et al.*, 2024 ▸). The present work aims at providing a definitive value for *x*, if any, by a successful attempt to determine the structure using the powder diffraction route since no single crystal is available.

## Experimental

### Powder diffraction

Two powder diffraction patterns of the commercial calcium bis(tetra­fluoro­borate) hydrate [Ca(BF_4_)_2_·*x*H_2_O, Alfa Aesar] were measured using a D501 Siemens Bragg–Brentano dif­frac­tometer, the sample being either pressed or dusted on the horizontal holder, showing strong differences due to preferred orientation (see Fig. S1 in the supporting information).

### Refinement

**Figure 1:**
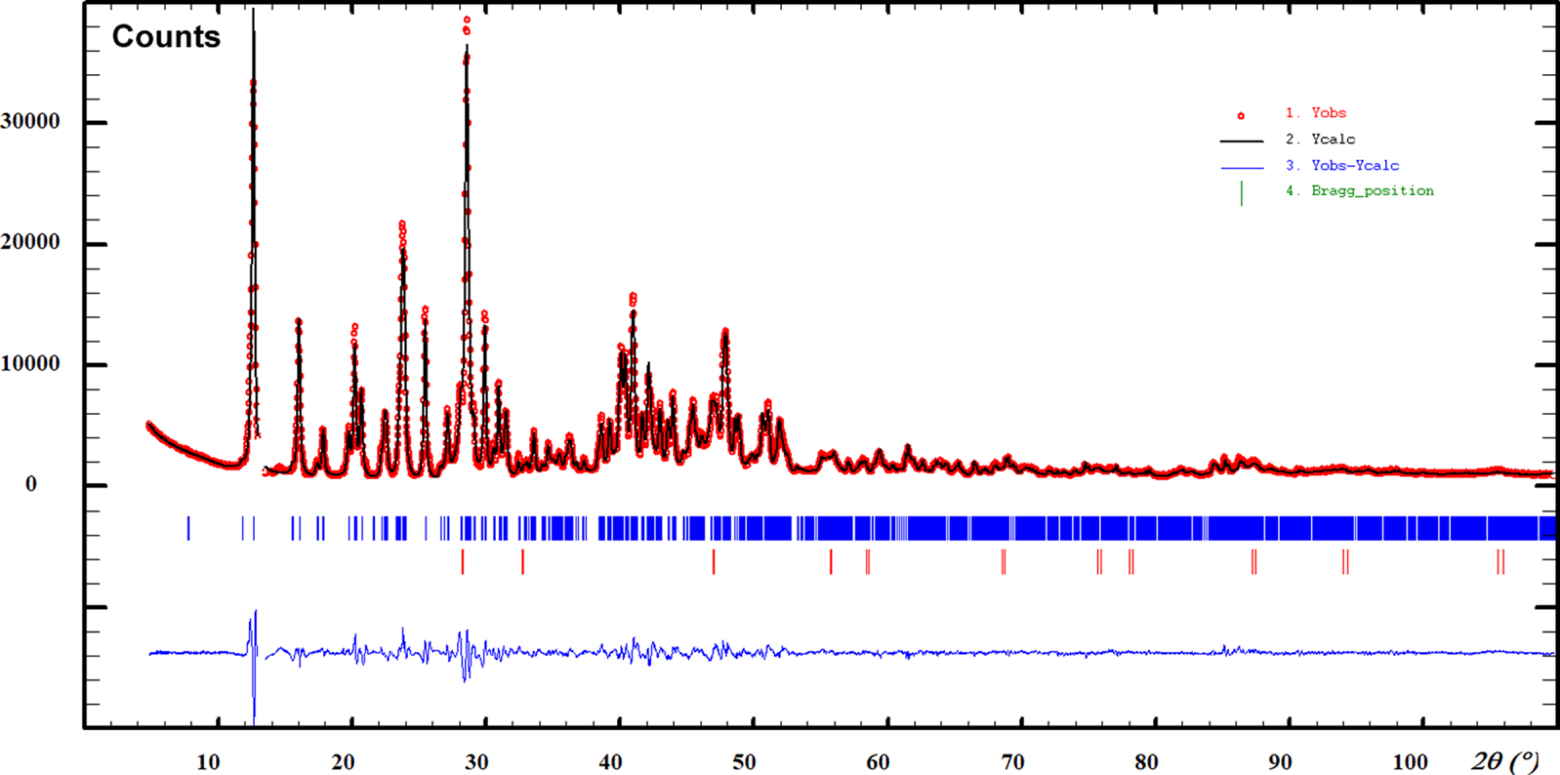
Refined diffraction pattern from laboratory data for Ca(H_2_O)_4_(BF_4_)_2_. Red dots represent the observed data and the black line represents the calculated pattern. Bragg ticks are the peak positions (main phase at the top and the CaF_2_ impurity below). The bottom blue curve shows the difference between the observed and calculated patterns. A peak close to 13° (2θ) which may correspond to the (002) reflection from one tiny single crystal of the anhydrous phase in diffraction position was removed by an excluded zone (see also Fig. S1 in the supporting information, showing another pattern where there is no such peak).

Indexing was realized using the *McMaille* software (Le Bail, 2004 ▸), leading to a triclinic cell. It was then confirmed and the intensities were extracted using the Le Bail method (Le Bail, 2005 ▸) implemented in the *FULLPROF* software (Rodríguez-Carvajal, 1993 ▸). The ortho­rhom­bic crystal structure of anhydrous Ca(BF_4_)_2_ (Jordan *et al.*, 1975 ▸) has a volume close to 1100 Å^3^ for *Z* = 8; one would expect *Z* = 2 for the hydrated phase having *V* ∼ 500 Å^3^. The direct-space *ESPOIR* software (Le Bail, 2001 ▸) provided a starting solution when using the [CaF_8_] square anti­prism taken from the anhydrous phase, moved randomly in the triclinic cell together with two B and five O atoms. In the resulting model, [BF_2_O_2_] tetra­hedra were formed inter­connecting [CaF_8_] anti­prisms in isolated infinite ribbons. After Rietveld (1969 ▸) refinements from this initial model, still using *FULLPROF*, it was concluded that *x* = 4; the initial [CaF_8_] block sharing four of its F corners with [BF_4_] tetra­hedra should be redefined as a [CaO_4_F_4_] square anti­prism. The hy­dro­gen-bonding scheme was then guessed observing the shortest distances between the O atoms and the terminal F atoms of the [BF_4_] tetra­hedra not in common with the calcium; six O—H⋯F and two O—H⋯O hy­dro­gen bonds were disclosed. During the final refinement, soft constraints were applied on the bonding scheme and on the [BF_4_] tetra­hedra. Scattering factors for B^3+^ cations were taken from Olukayode *et al.* (2023 ▸). The Rietveld plot is shown in Fig. 1 ▸. Crystal data, data collection and structure refinement details are summarized in Table 1 ▸.

## Results and discussion

**Figure 2:**
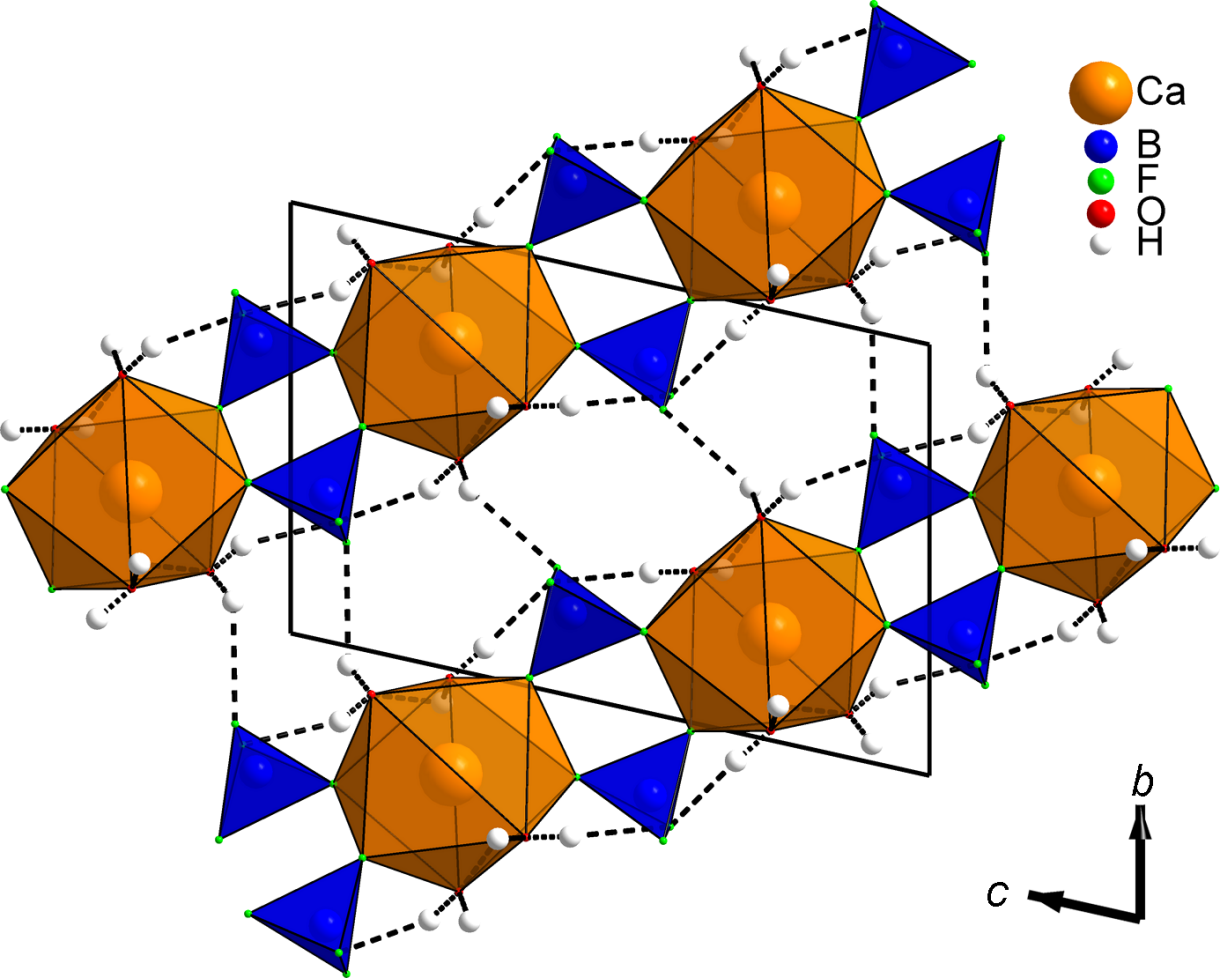
Unit-cell projection of the Ca(H_2_O)_4_(BF_4_)_2_ structure along the *a* axis, showing the [BF_4_] tetra­hedra in blue forming infinite ribbons extending along [011] by sharing half of their F corners with the [CaO_4_F_4_] square anti­prisms. This view shows mainly the O—H⋯F inter-ribbon bonding involving the terminal F atoms of the [BF_4_] tetra­hedra (not shared with Ca).

**Figure 4:**
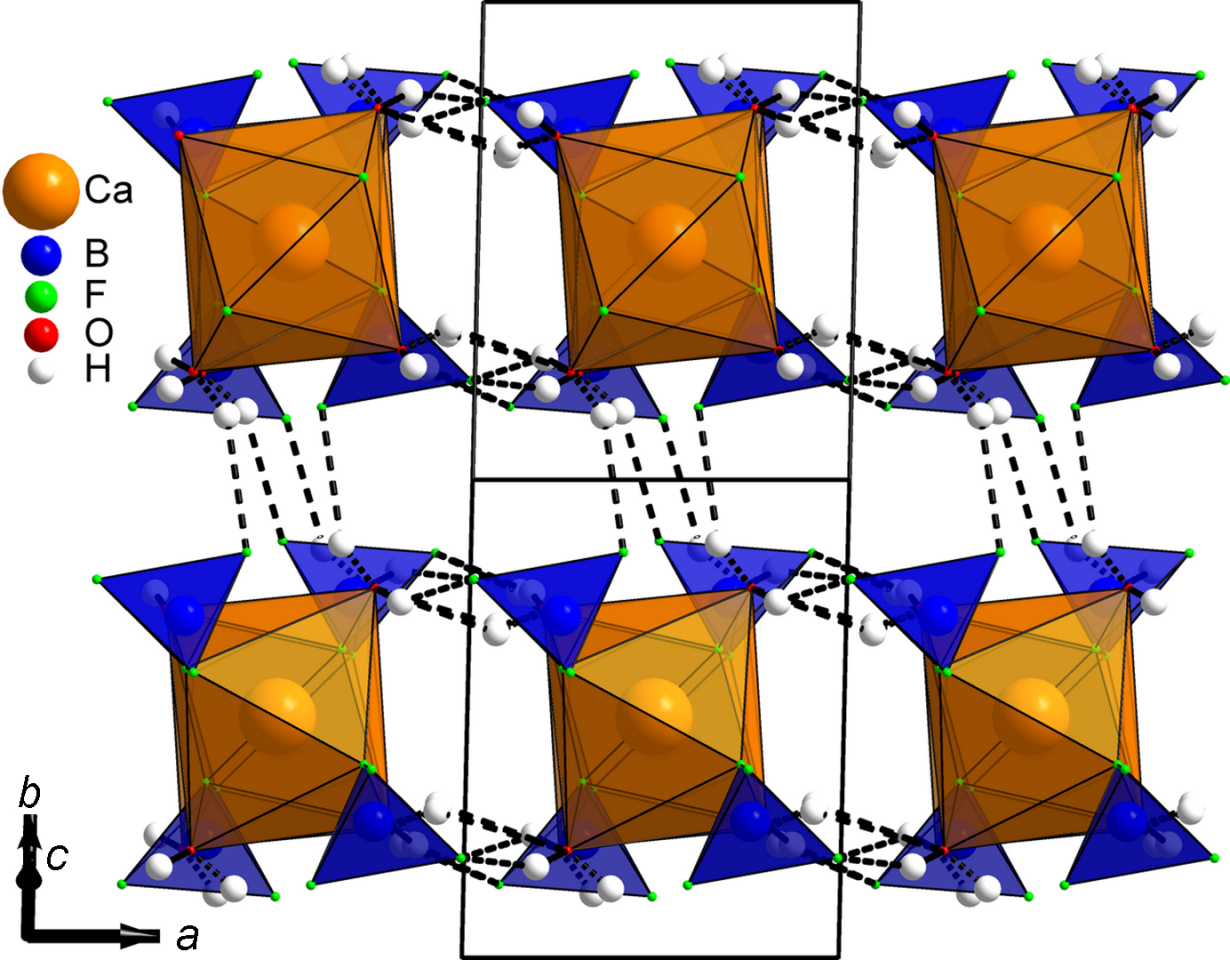
Unit-cell projection of the Ca(H_2_O)_4_(BF_4_)_2_ structure along the [0
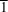
1] axis, showing the space between the 1D ribbons and how they are inter­connected by hy­dro­gen bonding.

Given that all four water mol­ecules are part of the [CaO_4_F_4_] square anti­prisms, the com­pound chemistry can be reformulated as Ca(H_2_O)_4_(BF_4_)_2_ instead of the previous Ca(BF_4_)_2_·(*x* = 4)H_2_O. Indeed, there is no place to acccomodate any additional water mol­ecule. Projections of the structure along the *a* and *b* axes are shown, respectively, in Figs. 2 ▸ and 3 ▸, disclosing the com­plex hy­dro­gen-bonding scheme inter­connecting ribbons built from the calcium in square anti­prisms sharing their F corners with [BF_4_] tetra­hedra (Table 2 ▸). A view in the direction of the ribbons (Fig. 4 ▸) shows how they are efficiently stacked. Six of the eight hy­dro­gen bonds are H⋯F pointing towards the terminal F atoms of the two BF_4_^−^ anions (F1, F2, F5 and F6, not shared with Ca), the remaining two hy­dro­gen bonds are H⋯O bonds involving atoms O3 and O4. There is no intra-ribbon hy­dro­gen bond in the structure. Each ribbon is inter­connected by hy­dro­gen bonding to four adjacent ribbons (Fig. 4 ▸), com­pleting the structure cohesion to three dimensions. It should be noticed that this bonding scheme is an hypothesis proposed from powder diffraction data, *i.e.* the H atoms do not come from a Fourier difference map. Then subtleties like bifurcated bonds are hardly seen; however, bond valence calculations in the supporting material are satisfying. Trying to explain the first step in the thermogravimetric analysis (TGA) corresponding closely to the departure of two water mol­ecules would be haza­rdous. Which two O atoms would first escape at 158 °C? A thermodiffractometry study would possibly reveal the existence of a dihydrate which could be formulated Ca(H_2_O)_2_(BF_4_)_2_. So the final model presented here would require either the production of large-enough single crystals or a neutron powder diffraction approach for com­plete confirmation, but the new Ca(H_2_O)_4_(BF_4_)_2_ formula looks likely. At least we definitely have a cell and the positions of the non-H atoms.

Searching ultimately for related materials, the title com­pound was finally found to be isostructural with calcium perchlorate tetra­hydrate, Ca(ClO_4_)_2_·4H_2_O (Hennings *et al.*, 2014*a* ▸), which is not unexpected. A search was made using the ‘tetra­hydrate’ keyword in all *Acta Crystallographica* articles, the calcium perchlorate tetra­hydrate appeared 49th in a list of 1313 papers. The unit-cell parameters of these two com­pounds are not close enough for obtaining a match from the *QualX* search-match sofware (Altomare *et al.*, 2015 ▸). Both phases present a similar hy­dro­gen-bonding scheme. In spite of Sr(BF_4_)_2_ being isostructural with Ca(BF_4_)_2_ (Goreshnik *et al.*, 2010 ▸), no strontium tetra­fluoro­borate tetra­hydrate was found in the literature; a trihydrate was characterized recently (Charkin *et al.*, 2023 ▸) and is tetra­gonal. Finally, Sr(ClO_4_)_2_·4H_2_O (Hennings *et al.*, 2014*b* ▸) is not isostructural with Ca(ClO_4_)_2_·4H_2_O; there is no ribbon and each perchlorate anion coordinates to a dimeric unit of two Sr^2+^ cations.

Anisotropy-induced physical properties are expected from such hy­dro­gen-bonded ribbons (Xia *et al.*, 2003 ▸), which is beyond the scope of the present article, but suggests it would be of inter­est to look more closely at the title com­pound and the perchlorate analog.

## Related literature

The following references are cited in the supporting information for this article: Brese & O’Keeffe (1991 ▸); Brown & Altermatt (1985 ▸).

## Supplementary Material

Crystal structure: contains datablock(s) global, I. DOI: 10.1107/S2053229625004395/vx3013sup1.cif

Rietveld powder data: contains datablock(s) I. DOI: 10.1107/S2053229625004395/vx3013Isup2.rtv

Comparison of powder patterns with and without preferred orientation. Search-match result. DOI: 10.1107/S2053229625004395/vx3013sup3.pdf

CCDC reference: 2451562

## Figures and Tables

**Figure 3:**
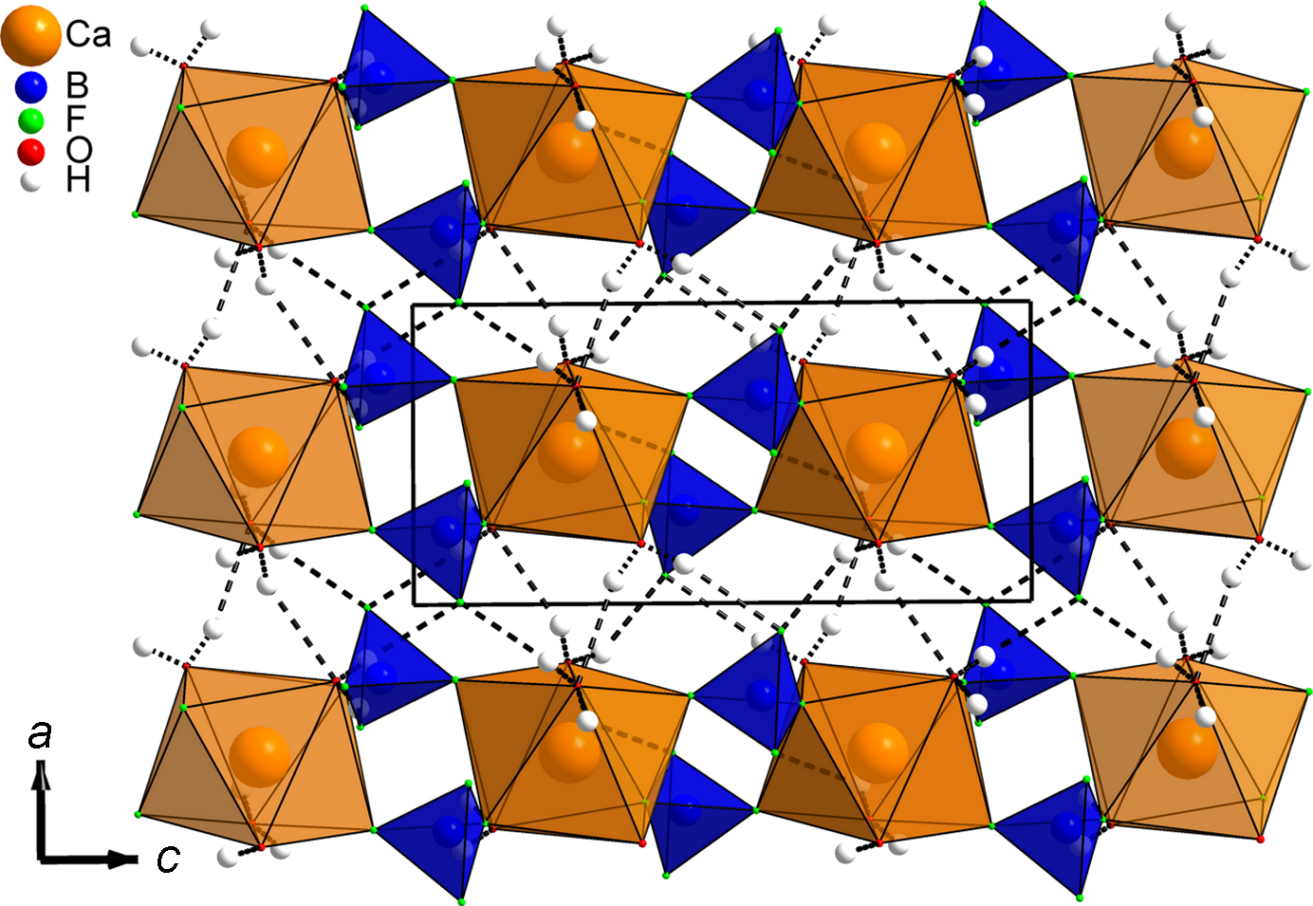
Unit-cell projection of the Ca(H_2_O)_4_(BF_4_)_2_ structure along the *b* axis, showing the intricate hy­dro­gen-bonding scheme, with both O—H⋯F and O—H⋯O hy­dro­gen bonds maintaining in 3D the 1D ribbons built from [CaO_4_F_4_] square anti­prisms sharing F corners with [BF_4_] tetra­hedra.

**Table 1:** Experimental details

Crystal data	
Chemical formula	Ca(BF_4_)_2_.4H_2_O
*M*_r_	285.76
Crystal system, space group	Triclinic, *P* 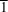
Temperature (K)	293
*a*, *b*, *c* (Å)	5.5192 (3), 7.6756 (3), 11.6518 (5)
α, β, γ (°)	77.439 (3), 89.579 (3), 88.625 (2)
*V* (Å^3^)	481.65 (4)
*Z*	2
Radiation type	Cu *K*α, λ = 1.540560 Å
Specimen shape, size (mm)	Flat sheet, 25 × 10

Data collection	
Diffractometer	Siemens D501
Specimen mounting	Plate sample holder
Data collection mode	Reflection
Scan method	Step
2θ values (°)	2θ_min_ = 4.817, 2θ_max_ = 109.817, 2θ_step_ = 0.020

Refinement	
*R* factors and goodness of fit	*R*_p_ = 6.213, *R*_wp_ = 8.419, *R*_exp_ = 1.973, *R*_Bragg_ = 3.78, χ^2^ = 18.207
No. of parameters	115
No. of restraints	56

Computer programs: *McMaille* (Le Bail, 2004 ▸), *ESPOIR* (Le Bail, 2001 ▸, *FULLPROF* (Rodríguez-Carvajal, 1993 ▸), *DIAMOND* (Brandenburg, 1999 ▸) and *publCIF* (Westrip, 2010 ▸).

**Table 2:** Hydrogen-bond geometry (Å, °)

*D*—H⋯*A*	*D*—H	H⋯*A*	*D*⋯*A*	*D*—H⋯*A*
O1—H11⋯O3^i^	0.84 (4)	2.19 (4)	2.859 (14)	138 (4)
O1—H12⋯F2^ii^	0.83 (7)	2.17 (6)	2.925 (14)	151 (5)
O2—H21⋯O4^iii^	0.86 (3)	2.23 (5)	3.015 (13)	152 (6)
O2—H22⋯F2^iv^	0.88 (2)	2.18 (3)	3.041 (12)	170 (7)
O3—H31⋯F5^v^	0.83 (5)	2.20 (4)	2.847 (12)	136 (4)
O3—H32⋯F6^vi^	0.82 (5)	2.07 (5)	2.845 (13)	156 (6)
O4—H41⋯F1	0.85 (4)	2.16 (4)	2.848 (13)	139 (4)
O4—H42⋯F6	0.86 (6)	2.05 (7)	2.873 (13)	161 (6)

Symmetry codes: (i) 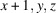; (ii) 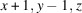; (iii) 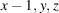; (iv) 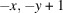, 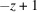; (v) 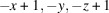; (vi) 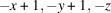.
