# Supplementary material for: Ca(BF4)2·xH2O redefined from powder diffraction as hy­dro­gen-bonded Ca(H2O)4(BF4)2 ribbons
Source: Acta Crystallogr C Struct Chem. 2025 May 19;81(Pt 6):338–41. doi: 10.1107/S2053229625004395 (PMC12138254; doi:10.1107/S2053229625004395)
Supplement: Supplementary file 3 [file c-81-00338-sup3.pdf]

STRUCTURAL CHEMISTRY

Volume 81 (2025)

Supporting information for article:

$Ca(BF_4)_2 \cdot xH_2O$ redefined from powder diffraction as hydrogen-bonded $Ca(H_2O)_4(BF_4)_2$ ribbons

Armel Le Bail

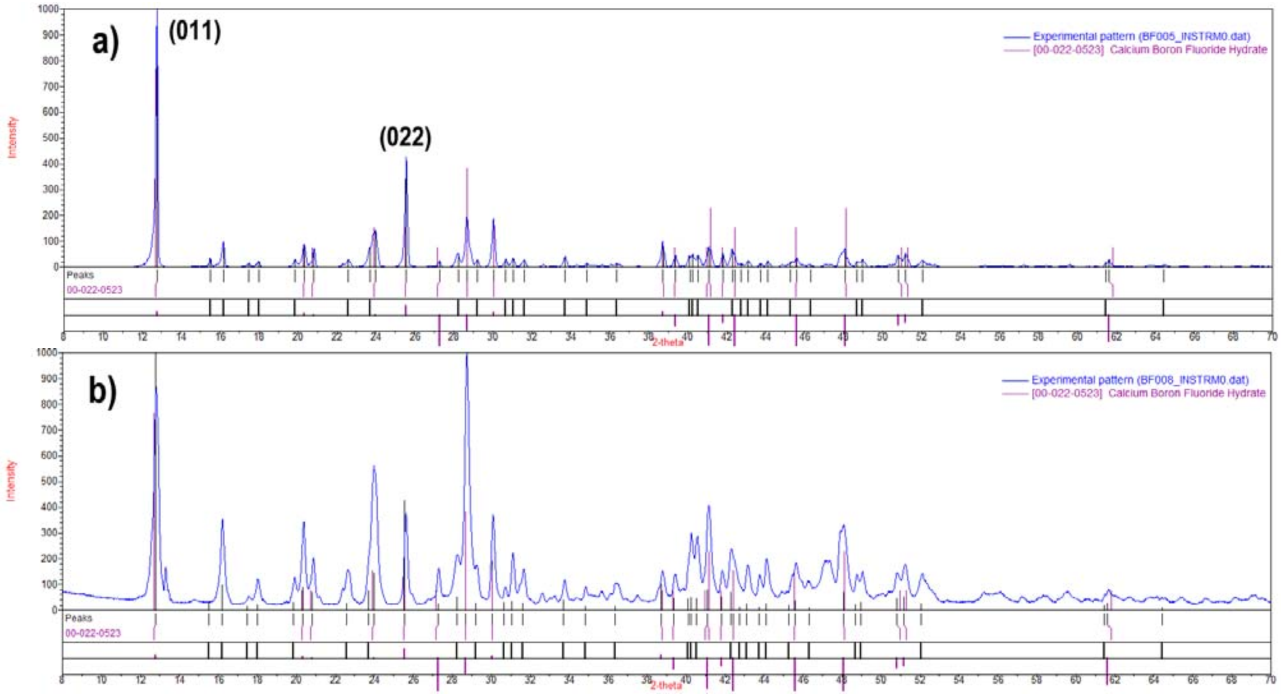

Fig. S1. Comparison between the X-ray powder patterns with preferred orientation (sample pressed) along [011] (*a*) and without (sample dusted through a sieve) (*b*). The PDF card 00-022-0523, calcium boron fluoride hydrate, is shown to match only with some of the most intense observed peaks (QUALX search-match software, Altomare *et al.*, 2015). The peak close to 13° (2theta) on (*b*) and unseen on (*a*) is at the (002) reflection position of $Ca(BF_4)_2$, the zone was excluded during the Rietveld refinements.
